# A Pleural Solitary Fibrous Tumor, Multiple Gastrointestinal Stromal Tumors, Moyamoya Disease, and Hyperparathyroidism in a Patient Associated with NF1

**DOI:** 10.1155/2015/375416

**Published:** 2015-09-13

**Authors:** Yoko Yamamoto, Ken Kodama, Shigekazu Yokoyama, Masashi Takeda, Shintaro Michishita

**Affiliations:** ^1^Department of Thoracic Surgery, Yao Municipal Hospital, Yao City, Osaka 581-0069, Japan; ^2^Department of Gastroenterological Surgery, Yao Municipal Hospital, Yao City, Osaka 581-0069, Japan; ^3^Department of Pathology, Yao Municipal Hospital, Yao City, Osaka 581-0069, Japan; ^4^Department of Breast and Endocrine Surgery, Osaka University, Osaka 565-0871, Japan

## Abstract

Neurofibromatosis type 1 (NF1), also called von Recklinghausen's disease, is a multisystemic disease caused by an alteration of the NF1 gene, a tumor suppressor located on the long arm of chromosome 17 (17q11.2). Loss of the gene function, due to a point mutation, leads to an increase in cell proliferation and the development of several tumors. We report a 60-year-old female patient manifesting hypercalcemia due to hyperparathyroidism, a solitary fibrous tumor (SFT) of the pleura, multiple gastrointestinal stromal tumors (GISTs), and moyamoya disease associated with NF1. The SFT and GISTs were removed by staged operations. Then, hypercalcemia was successfully controlled after resection of the parathyroid adenoma. Based on a literature review, these combinations have never been reported, and the relevant literature is briefly discussed.

## 1. Introduction

Neurofibromatosis type 1 (NF1) is an autosomal dominant inherited disease characterized by café-au-lait spots and multiple dermal neurofibromatosis. This condition is also known as von Recklinghausen's disease. NF1 is caused by mutation of the NF1 gene, which spans over 350 kb of genomic DNA on chromosome 17q11.2. The protein encoded by the NF1 gene is neurofibromin, which is a member of the GTPase-activating protein (GAP) family of Ras regulatory proteins.

NF1 is also associated with several tumors. Some reports have described the association of NF1 and GISTs, which are the most common mesenchymal neoplasms of the gastrointestinal tract. In many cases of NF1, multiple GISTs predominantly involve the small intestine. On the other hand, a solitary fibrous tumor (SFT) is a rare spindle cell neoplasm, usually occurring in the pleura. The association between an SFT and NF1 has not been elucidated. Moyamoya disease, which is a cerebrovascular disease of unknown cause, is also rarely seen in NF1 patients.

The association of NF1 and primary hyperparathyroidism is also described as a rare entity. This association supports the hypothesis that it is one of the variant types of multiendocrine neoplasia (MEN) syndrome.

Here, we report the results of treating an NF1 patient with the coexistence of multiple GISTs of the gastrointestinal tract, SFT of the pleura, hyperparathyroidism, and moyamoya disease.

## 2. Case

In August, 2013, a 60-year-old Japanese female consulted her primary physician for melena. Her laboratory tests showed anemia and hypercalcemia. Whole body computed tomography (CT) performed at that time showed an anterior mediastinal nodule, and an abdominal tumor was detected concomitantly. Then, she was referred to our hospital for further investigation.

Physical examination revealed café-au-lait spots as well as multiple skin nodules distributed over her entire body and Lisch nodules on her eyes, thus showing typical features of NF1. Her two daughters were positive for similar skin findings, but there was no evidence of familial MEN syndrome.

Laboratory tests showed a serum calcium level of 11.8 mg/dL (normal range: 8.6–10.1), a phosphorus level of 2.4 mg/dL (normal range: 2.5–4.5), and a plasma intact parathyroid hormone (PTH) level of 427.2 pg/mL (normal range: <65.0), and a diagnosis of primary hyperparathyroidism was made. No tumor markers were elevated.

**Figure 1:**
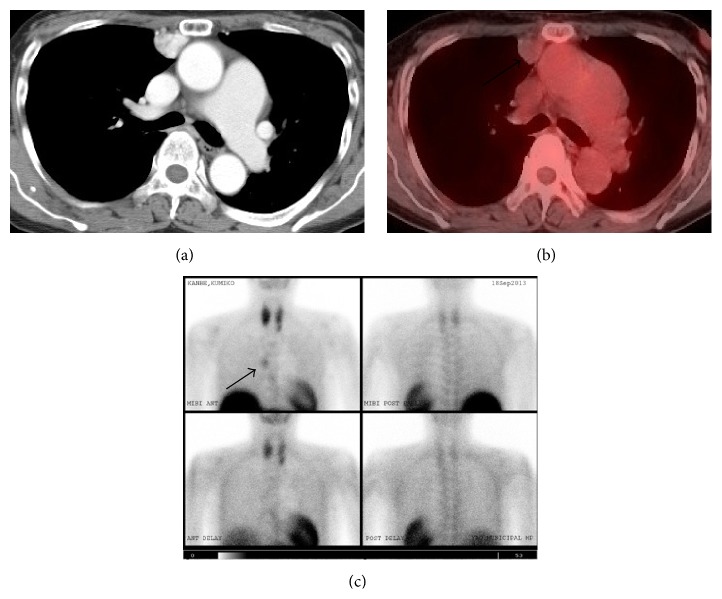
Mediastinal nodule (arrow). (a) CT revealed the presence of a 20 mm nodule in the anterior mediastinum. (b) PET-CT imaging revealed FDG uptake in the nodule. (c) Tc 99m MIBI scan showing an intense focus in the anterior mediastinum.

**Figure 2:**
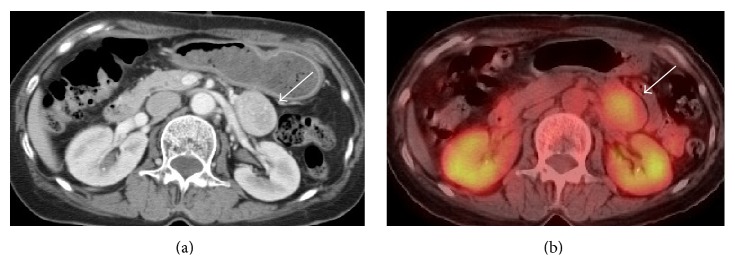
Abdominal mass (arrow). (a) CT revealed a 40 mm, well-circumscribed, firm, and well-enhanced mass adjacent to the upper jejunum. (b) PET-CT imaging showing the abdominal mass with an SUV max of 4.2.

Her chest CT revealed the presence of a 20 mm nodule in the anterior mediastinum adjacent to the right pleura (Figure 1(a)). The nodule showed no invasion to the surrounding structures. Abdominal CT revealed a 40 mm, well-circumscribed, firm, and well-enhanced mass adjacent to the upper jejunum (Figure 2(a)). Positron emission tomography (PET) revealed focal FDG uptake (standard uptake value (SUV) max, 4.2) in the abdominal mass (Figure 2(b)) but not mediastinal nodule (Figure 1(b)). Tc 99m MIBI parathyroid scintigraphy demonstrated an intense focus in the anterior mediastinum (Figure 1(c)). Thus, the mediastinal nodule was diagnosed as an ectopic parathyroid adenoma. Magnetic resonance imaging (MRI) of the abdomen demonstrated the mass showing a low signal intensity in T1-weighted images and intermediate signal intensity in a T2-weighted image.

Both gastroscopic and colonoscopic examinations were within their normal limit. Endoscopic ultrasonography (EUS) demonstrated that the internal echo of the main lesion was slightly heterogeneous and separated from the pancreas. Spindle cells were verified by EUS-fine needle aspiration (EUS-FNA) cytology. Based on these findings, the abdominal tumor was suspected to be a GIST.

**Figure 3:**
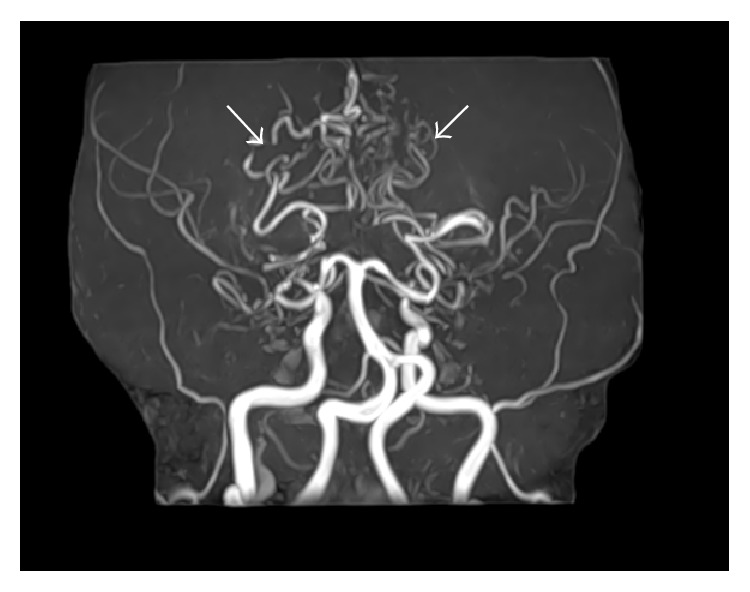
MR angiography confirmed occlusion of the bilateral internal carotid artery (ICA) and the absence of the anterior and middle cerebral arteries with multiple tiny basal collateral arteries (arrows).

Further examinations using MRI were conducted to investigate the clinical manifestations of NF1. As a result, brain MRI revealed no brain tumor. On the other hand, MR angiography showed occlusion of the bilateral internal carotid artery (ICA) and the absence of the anterior and middle cerebral arteries with multiple tiny basal collateral arteries (Figure 3). These findings are consistent with moyamoya disease.

**Figure 4:**
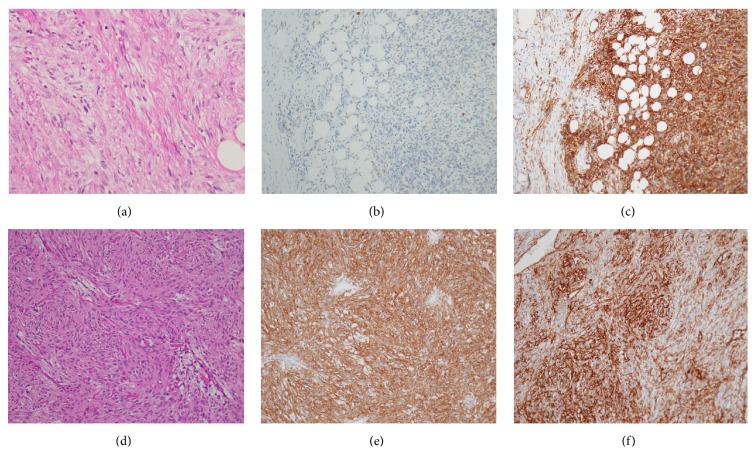
Histologic findings. A SFT composed of a solid unorganized proliferation of spindle cells with small and mildly irregular nuclei (a) (hematoxylin and eosin; ×40), which showed the absence of c-Kit (b) and the presence of CD34 (c) markers on immunohistochemical staining (×20). A GIST composed of spindle-shaped cells with marked cellularity (d) (hematoxylin and eosin; ×40), which showed the presence of c-Kit (e) and CD34 (f) markers on immunohistochemical staining (×20).

Initially, we attempted resection of the anterior mediastinal nodule diagnosed with ectopic parathyroid adenoma to control her hypercalcemia. We resected the nodule with hemithymus through a median sternotomy. However, after the operation, the serum calcium level was not decreased. Based on permanent section histology (Figure 4(a)), the tumor was composed of a solid, unorganized proliferation of spindle cells with small and mildly irregular nuclei. No significant necrosis was seen. Immunohistochemistry examination shows positive reactivity for c-Kit (Figure 4(b)), CD34 (Figure 4(c)), bcl-2, and STAT6, but it shows negative result for AE1/3, S-100 protein, and SMA. The Ki-67 labeling index was irregularly expressed, exhibiting positivity in up to about 5% of neoplastic cell. There was neither cell invasion in the thymus nor evidence of malignancy. Pathologically, the tumor was diagnosed as a benign SFT of the pleura based on the above findings. Her postoperative course was uneventful. She was discharged with no complications under medical control of hypercalcemia.

Three months after the first operation, she received a laparotomy under a clinical diagnosis of GIST of the upper jejunum. The operative findings showed multiple mucosal tumors in the stomach, 2nd portion of the duodenum, distal to the ligament of Treitz, and in the upper part of the jejunum. During the operation, we found 9 tumors, and all of them were removed. These tumors showed an extramural growth pattern, the main tumor measured 35 mm in diameter distal to the ligament of Treitz and the other tumors were less than 10 mm. Histological examination (Figure 4(d)) showed that the tumors were highly cellular and composed of spindle-shaped cells arising in the proper muscle layer of the gastrointestinal wall. No significant necrosis was seen. The overlying mucosa was intact. Immunohistochemically, the tumors were strongly stained positive for c-Kit (Figure 4(e)) and CD34 (Figure 4(f)), whereas S-100 and SMA were negative. The Ki-67 labeling index was less than 2-3%. The postoperative course after the second operation was also uneventful, and she was discharged with no complications.

Four months after the second operation, we performed MIBI scintigraphy again. The second time, MIBI scintigraphy revealed focal uptake in the left lower pole of thyroid gland and neck ultrasound showed solid nodule in the left lower pole of thyroid gland. She subsequently underwent parathyroidectomy to control hypercalcemia. The pathological diagnosis was parathyroid adenoma. After the operation, the value of serum Ca rapidly decreased to within the normal range. She showed a favorable condition 14 months after the first surgery without symptoms.

## 3. Discussion

The NF1 occurs in about 1 in 3,000–3,500 births, and it can be familial with an autosomal inheritance pattern. In addition to cutaneous café-au-lait spots and multiple neurofibromas, various accompanying lesions are known to occur in the eyes, bone, central nerves, and endocrine system [1, 2]. Gastrointestinal abnormalities in NF1 patients have been reported to occur in up to 10–25% of patients, including mesenchymal neoplasms, neuroendocrine tumors of the duodenum, hyperplasia of intestinal neural tissues, and other gastrointestinal neoplasms [3]. The overall rate of NF1 among GIST patients can reach up to 6% [4]. GISTs originate from the intestinal cells of Cajal (ICC). They are often found in patients with anemia, constipation or obstruction, and palpation of a tumor. Making an early diagnosis of a GIST is important due to the risk of malignancy and hemorrhagic-obstructive complications. GISTs can be better defined on immunohistochemical examination: positive activity for c-Kit and CD34 and negative activity for S-100 [5]. GISTs are the most common mesenchymal neoplasms of the gastrointestinal tract and they have been described in association with NF1. The incidence of GISTs in NF1 patients varies from 3.9 to 25% [4]. The majority of GISTs in NF1 patients were reported to be multicentric and mainly localized in the jejunum, as in the present case, and these tumors were not associated with malignancy, suggesting a favorable long-term prognosis, which is rarely observed in sporadic GISTs. Surgical resection remains the mainstay of treatment and offers the only chance for cure [6, 7]. Adjuvant chemotherapy or radiotherapy had not been proven to be effective [6].

A solitary fibrous tumor (SFT) is an uncommon mesenchymal neoplasm that arises primarily from the pleura. Currently, SFTs are immunohistochemically characterized by negative activity for cytokeratin, suggestive of an epithelial origin, and by positive reactivity for CD34, suggestive of a mesenchymal origin, and they are considered to arise from undifferentiated mesenchymal cells in the subpleural connective tissue. The major difference between GISTs and SFTs was strong c-Kit immunoexpression in all GISTs and the absence of this expression in all SFTs [5]. Recently, a recurrent gene fusion NAB2-STAT6 has been identified as molecular hallmark. Molecular detection of the fusion gene and immunohistochemical expression of nuclear STAT6 can be helpful in diagnosing SFT [8]. Adequate therapy consists of complete resection. Histopathologically, SFTs are classified into benign and malignant forms. The cell density, necrosis, number of mitotic figures, and cell atypia indicate malignancy [9]. Although surgical resection is the treatment rule for pleural SFTs, many SFTs were reported to recur even if they had been diagnosed as benign [10]. Thus, SFTs require complete surgical resection and careful long-term follow-up even if benign. According to our search, the association between SFTs and NF1 has been reported only once before in the English language literature. Conzo et al. firstly described a right suprarenal SFT in a patient with NF1. However, NF1 gene mutation has not been investigated systematically in SFT cases, and the available evidence in the literature is insufficient [11]. Our case is the second reported case of the coexistence of NF1 and SFT. In our case, SFT mimicked the appearance of ectopic parathyroid adenoma based on the finding of Tc-99m MIBI parathyroid scintigraphy. Tc-99m MIBI, a lipophilic cationic molecule which was initially used for cardiac imaging, showed uptake in the mitochondria and cytoplasm of parathyroid tissue. The distribution of Tc-99m MIBI is proportional to blood flow and mitochondrial activity. The sensitivity and positive predictive values of Tc-99m MIBI are 82.1 and 93%, respectively. False uptake of Tc-99m MIBI has been documented in benign and malignant tissues with a high mitochondrial content. Most cases of false-positive Tc-99m MIBI involve thyroid disease. However, other tissues, such as lung, brain, and bone, and carcinoid tumors, lymphoma and thymoma, can also produce false-positive results [12].

Moyamoya disease is characterized by progressive stenosis or occlusion at the distal ends of the bilateral carotid arteries that may subsequently progress to their major branches. The clinical findings present with neurological symptoms, whereas ischemic stroke develops in young adults and subarachnoid hemorrhage develops in older patients in moyamoya disease. Most cases are asymptomatic, as in our present case. The prevalence of moyamoya disease in NF1 patients is estimated at 0.6%, among more than one hundred cases reported in pediatric patients [2, 13]. The gene abnormality has been detected in chromosome 17q25.2, which is in close proximity to the NF1 gene on chromosome 17q11.2. There are several reports concerning the association of moyamoya disease and NF1, which could be explained by the close proximity of genes on chromosome 17 [14].

A combination of hyperparathyroidism and NF1 is also a rare phenomenon. Although some NF1 patients who developed primary hyperparathyroidism have been reported in the literature, the pathogenesis of parathyroid adenoma in NF1 patients has not been yet elucidated, but multiple endocrine adenoma and a genetic link have been suggested. Daly et al. reported that their patients' conditions resembled Sipple's syndrome in that they had both parathyroid adenoma and neurofibromatosis, which suggested the tumors to be genetically linked [15]. Gkaliagkousi et al. described a case with a clinically and genetically established NF1 diagnosis and clinically established MEN2A diagnosis, but genetic testing for MEN2A was negative [16]. Also, there are several case reports in which NF1 was combined not only with the presence of parathyroid neoplasms, but also with other neoplastic disorders [17]. This association supports the hypothesis of a variant of MEN syndrome.

NF1 has been reported to be associated with a number of neoplasms; however, to the best of our knowledge, the coexistence of NF1, SFT, GIST, and moyamoya disease has not been previously reported. Therefore, this case is likely to be the first reported case to involve the coexistence of all four conditions. Further studies are needed to elucidate the association between these rare but interesting conditions. In conclusion, we encountered pleural SFT, multiple GISTs, moyamoya disease, and hyperparathyroidism in a patient with NF1.
